# Low-cost rapid detection of rifampicin resistant tuberculosis using bacteriophage in Kampala, Uganda

**DOI:** 10.1186/1476-0711-6-1

**Published:** 2007-01-09

**Authors:** Hamidou Traore, Sam Ogwang, Kim Mallard, Moses L Joloba, Francis Mumbowa, Kalpana Narayan, Susan Kayes, Edward C Jones-Lopez, Peter G Smith, Jerrold J Ellner, Roy D Mugerwa, Kathleen D Eisenach, Ruth McNerney

**Affiliations:** 1 London School of Hygiene & Tropical Medicine, Keppel Street, London, WC1E 7HT, UK; 2 Joint Clinical Research Centre, Plot 893, Ring Road, Butikiro House, Mengo, P.O. Box 10005, Kampala, Uganda; 3 Makerere University Medical School, Mulago Hospital, Kampala, Uganda; 4 New Jersey Medical School, University of Medicine and Dentistry of New Jersey, Newark, New Jersey, USA; 5 University of Arkansas for Medical Sciences, Little Rock, Arkansas, USA

## Abstract

**Background:**

Resistance to anti-tuberculosis drugs is a serious public health problem. Multi-drug resistant tuberculosis (MDR-TB), defined as resistance to at least rifampicin and isoniazid, has been reported in all regions of the world. Current phenotypic methods of assessing drug susceptibility of *M. tuberculosis *are slow. Rapid molecular methods to detect resistance to rifampicin have been developed but they are not affordable in some high prevalence countries such as those in sub Saharan Africa. A simple multi-well plate assay using mycobacteriophage D29 has been developed to test *M. tuberculosis *isolates for resistance to rifampicin. The purpose of this study was to investigate the performance of this technology in Kampala, Uganda.

**Methods:**

In a blinded study 149 *M. tuberculosis *isolates were tested for resistance to rifampicin by the phage assay and results compared to those from routine phenotypic testing in BACTEC 460. Three concentrations of drug were used 2, 4 and 10 μg/ml. Isolates found resistant by either assay were subjected to sequence analysis of a 81 bp fragment of the *rpo*B gene to identify mutations predictive of resistance. Four isolates with discrepant phage and BACTEC results were tested in a second phenotypic assay to determine minimal inhibitory concentrations.

**Results:**

Initial analysis suggested a sensitivity and specificity of 100% and 96.5% respectively for the phage assay used at 4 and 10 μg/ml when compared to the BACTEC 460. However, further analysis revealed 4 false negative results from the BACTEC 460 and the phage assay proved the more sensitive and specific of the two tests. Of the 39 isolates found resistant by the phage assay 38 (97.4%) were found to have mutations predictive of resistance in the 81 bp region of the *rpo*B gene. When used at 2 μg/ml false resistant results were observed from the phage assay. The cost of reagents for testing each isolate was estimated to be 1.3US$ when testing a batch of 20 isolates on a single 96 well plate. Results were obtained in 48 hours.

**Conclusion:**

The phage assay can be used for screening of isolates for resistance to rifampicin, with high sensitivity and specificity in Uganda. The test may be useful in poorly resourced laboratories as a rapid screen to differentiate between rifampicin susceptible and potential MDR-TB cases.

## Background

The emergence of drug resistant strains of *Mycobacterium tuberculosis *is of growing concern. Multi-drug resistant disease (MDR-TB), where the strain is resistant to both the major anti-tuberculosis drugs rifampicin and isoniazid, has been reported in all regions of the world. Incidences of MDR exceeding 10% of TB caseloads have been reported in parts of Central Asia, China, Eastern Europe, Russia and Africa [1]. The prognosis of patients with MDR-TB is poor and, unless alternative anti-tuberculosis drugs are administered, they are likely to remain infectious until death. Treatment of MDR-TB is both expensive and difficult to administer as it requires prolonged treatment of at least 18 months with 'second line' drugs that exhibit enhanced toxicity Early detection of MDR-TB is important not only for the patient, but also to limit transmission and the spread of drug resistant disease. Traditional phenotypic methods of detecting drug resistant disease are slow due to the protracted growth rate of *M. tuberculosis*, with results often taking weeks to obtain. Rapid molecular methodologies have been developed that detect mutations predictive of resistance to rifampicin. These tests examine the *rpo*B gene encoding the β-subunit of bacterial DNA dependent RNA polymerase and they have been demonstrated to have high accuracy for detecting resistant strains of *M. tuberculosis *[2]. In some settings resistance to rifampicin is highly predictive of MDR-TB [3] and these rapid tests may be used to investigate suspected MDR-TB cases or to monitor high risk patients such as those failing standard treatment regimens. However, the new molecular technologies have not been implemented in resource limited settings due to their high cost and the requirement for specialist skills and equipment. Most high prevalence countries continue to use slow culture-based methods to investigate suspected MDR-TB cases and new simple, rapid tests are needed that are affordable in these settings.

We have previously described a 'low-tech' rapid method for investigating the susceptibility of *M. tuberculosis *to rifampicin that uses mycobacteriophage D29 [4]. In this technology mycobacteriophages are allowed to infect the bacteria, successful replication and production of progeny phage being indicative of the presence of viable mycobacteria. Rifampicin disrupts phage replication by preventing synthesis of bacterial mRNA and when critical concentrations of this drug are present progeny phage will only be observed in those strains resistant to the drug [5]. A microwell plate version of this technology has been developed which allows high-throughput screening of *M. tuberculosis *isolates [6]. To assess the performance of this simple test in a low-income, high prevalence country, the method was transferred to a TB laboratory in Uganda. The study was part of a multidisciplinary project aimed at developing strategies for the management of MDR-TB in the Kampala region. A panel of stored strains was selected to undergo blinded testing by the phage assay. Results were compared to those obtained by BACTEC 460 system (Becton Dickinson, Sparks, Maryland, USA); a broth based phenotypic method routinely employed in this laboratory. Strains identified as resistant to rifampicin by either assay were investigated for mutations in an 81 bp segment of the *rpo*B gene by sequencing [7]. Strains with discordant susceptibility test results were investigated further by application of a second phenotypic test to assess minimal inhibitory concentrations of the drug. To appraise the usefulness of the phage assay in this setting we also assessed the rapidity of the test and cost of the reagents.

## Methods

All manipulation of live *M. tuberculosis *was performed under biohazard category 3 safety conditions using a microbiological safety cabinet in accordance with local regulations. One hundred and forty-nine cultures of *M. tuberculosis *isolated from 129 patients were selected for testing at the Mycobacteriology Laboratory of the Joint Clinical Research Centre (JCRC) in Kampala, Uganda. Strains for the study were selected from stored cultures previously isolated from subjects enrolled into several IRB-approved studies at the Tuberculosis Research Unit in Uganda. Prior to testing all isolates were freshly sub-cultured on Middlebrook 7H10 agar or Lowenstein-Jensen (LJ) medium. *M. tuberculosis *H37Rv was used as a susceptible control strain and *M. tuberculosis *TMC 331 as a resistant control strain. All strains were subjected to testing for susceptibility to rifampicin by the phage assay and a traditional phenotypic test. Statistical analysis of results was performed using STATA 9.0 (Texas, USA)

### Phage assay

The details of the assay and production of phage D29 have been described previously [8]. Indicator plates for detection of progeny phage were prepared by adding 10% v/v of a stationary phase culture of *M. smegmatis *mc^2^155 to 1.5% agar in Luria-Bertani broth (Difco, Becton Dickinson, Sparks, USA.). Rifampicin was prepared from a stock solution of 20 mg/ml in dimethylformamide (Sigma-Aldrich, Poole, UK). 75 μl of drug at 4, 8 and 20 μg/ml were placed to the wells of a sterile 96-well plate (Greiner Labortechnik, Stonehouse, UK) to give a final working concentration of 2, 4 and 10 μg/ml. Aliquots containing no drug were also plated.

Bacterial suspensions of isolates under test were prepared from growth on solid medium in 2 ml of Luria-Bertani broth supplemented with 1 mM calcium chloride (assay broth). Samples were vortexed in the presence of glass beads to disaggregate clumps and left to stand for at least 5 min to allow aerosols to settle. Aliquots of 75 μl were placed in each well of the microwell plate containing drug solutions. The plate was then covered, sealed in a plastic bag and incubated at 37°C for 24 hours. D29 phage suspension was diluted in the assay broth and an aliquot of 50 μl added to each well, giving a final concentration of 2.5 × 10^7 ^phage/ml. The plate was resealed and incubated at 37°C for 90 min. Aliquots of 100 μl of freshly prepared solution of 30 mM ferrous ammonium sulphate (Sigma-Aldrich, Poole, UK) were added to each well and mixed by pipetting. Aliquots of 10 μl from each well were then spotted onto the surface of the *M. smegmatis *indicator plate. After absorption of drops in the agar medium the plates were sealed in plastic bags and incubated overnight at 37°C. The number of plaque forming units (pfu) was recorded on the drug-containing assay and compared with the number of pfu on the drug-free control. An isolate was recorded as susceptible when no pfu were observed from drug containing samples and as resistant when pfu were observed for the assay corresponding to each drug concentration. The assay and interpretation of results was performed blinded, without prior knowledge of the outcome of other susceptibility tests.

### Phenotypic susceptibility testing

Drug susceptibility testing was performed using the BACTEC 460 system (Becton Dickinson, Sparks, Maryland, USA) following the manufacturer's recommendations [9]. One standard drug concentration (2 μg/ml) was reconstituted from lyophilized drugs supplied by the manufacturer. The test inoculates were standardized prior to the addition of 0.1 ml volumes to the vials containing drug, and to a no drug control. Samples were incubated at 37°C and daily readings were taken and interpretation performed after comparing the changes in the growth indices of the inoculated control with that of the test drug.

### Nucleotide sequencing

Strains found resistant by either by the BACTEC or the Phage assay were investigated for mutations in a region of the *rpo*B gene. Sequencing was performed on in the Genome Research Centre at the London School of Hygiene & Tropical Medicine. DNA was extracted from cells grown on Middlebrook 7H11 agar using the CTAB (hexadecyl trimethyl ammonium bromide)-NaCl method described by Van Embden and collaborators [10]. A 255-bp fragment of the *rpo*B gene including the 81-bp core region was amplified by PCR using primers RP4T (5'-GAG GCG ATC ACA CCG CAG ACG T-3') and RP8T (5'-GAT GTT GGG CCC CTC AGG GGT T-3') [11] and a two-step amplification programme (3 min at 95°C followed by 30 cycles of [95°C for 30s; 65°C for 45s and 72°C for 1 min]). PCR products were purified through QIAquick PCR Purification columns (Qiagen, Crawley, UK). Sequencing of both forward and reverse strands of the amplicons was performed using the amplification primers (RP4T and RP8T) and the BigDye Terminator Cycle Sequencing kit v.3.1. (Applied Biosystems, Foster City, CA, USA). The sequences obtained were compared to wild type *M. tuberculosis *H37Rv RNA-polymerase beta subunit (*rpo*B) gene (partial cds U12205) using DNAStar MegAlign programme (DNAStar, Wisconsin, USA).

### Minimal inhibitory concentration

Minimal inhibitory concentrations (MIC) were determined using the assay described by Abate *et al *[12]. This assay is based on detection of bacterial growth by a redox dye 3-(4,5-dimethylthiazol-2-yl)-2,5-diphenyltetrazolium bromide (MTT) (Sigma-Aldrich, Poole, UK). Serial two-fold dilutions of rifampicin were prepared in the wells of a sterile flat bottom 96-well plate (Greiner Labortechnik, Stonehouse, UK) to achieve concentrations ranging from 0.4 – 100 μg/ml in 100 μl Middlebrook 7H9 broth supplemented with OADC (Becton Dickinson, Sparks, USA). The inoculum was prepared from fresh Lowenstein Jensen cultures in Middlebrook broth and 100 μl of bacterial suspension added to each well. A growth control well containing no drug was included for each isolate. After seven days of incubation at 37°C, 10 μl of the MTT solution (5 mg/ml) was added to each well and the plate was re-incubated overnight. Aliquots of 50 μl of formazan solubilising buffer [1:1 (v/v) 20% SDS: 50% *N*,*N*-dimethylformamide (Sigma, Poole, UK)] were then added to the wells and the plate re-incubated for three hours. A change in colour from yellow to violet indicated growth of bacteria and the MIC was interpreted as the lowest concentration that inhibited bacterial growth.

## Results

### Susceptibility to rifampicin

Of the 149 isolates tested by BACTEC 114 were found susceptible and 35 resistant to rifampicin at 2 μg/ml. The number of isolates found susceptible by the phage assay varied with the concentration of drug used. (Table 1). When tested at a drug concentration of 2 μg/ml 91 isolates were found susceptible and 58 resistant. Twenty three isolates found resistant by the phage assay at this drug concentration were found susceptible by BACTEC. However, when concentration of rifampicin was increased to 4 or 10 μg/ml the number of strains found resistant by the phage assay dropped to 39 and, when compared to BACTEC, four isolates with discordant results were recorded. The 35 isolates found resistant by BACTEC were also found resistant by the phage assay at all drug concentrations tested. Comparison of the phage assay when used at 4 or 10 μg/ml with the BACTEC provided an overall agreement for the two tests of 97.3% (n = 145/149, 95% CI; 93.3–99.3%). If the results from BACTEC are taken as the "gold standard" for assessing resistance and susceptibility, the sensitivity of the phage test was 100% (i.e. 35/35: lower 97.5% one sided CI = 90%) and the specificity of the phage test was 96.5% (i.e. 110/114: 95% CI 91.3–99.1%).

**Table 1:** Susceptibility of isolates tested by BACTEC 460 and Phage assay.

Assay	Drug concentration μg/ml	Susceptible Isolates	Resistant isolates
BACTEC 460	2	114	35*
Phage	2	91	58
Phage	4	110	39
Phage	10	110	39

* All 35 isolates found resistant by BACTEC 460 were also found to be resistant by the phage assay (at each concentration).

The statistical significance of the difference between the two assays can be assessed by comparing the number of discrepant results. For 4 isolates the phage assay indicated resistance and the BACTEC indicated susceptibility and there were no isolates which were susceptible by the phage test and resistant by BACTEC. This difference (4 vs 0) is not statistically significant. However, further genetic analysis (see below) suggested that the phage assay might be the more sensitive of the two. Phage assay results were available within 48 hours.

### Mutation analysis

Sequencing of the *rpo*B core region was performed on the 39 isolates found resistant by the phage assay at 4 or 10 μg/ml and on 18 of the 19 isolates found resistant to 2 μg/ml but susceptible at 4 or 10 μg/ml.

All but one of the 39 isolates found resistant at 4 or 10 μg/ml in the phage assay had a *rpoB *mutation associated with RMP resistance (Table 2). A double mutation was found in two isolates. The isolate with no mutation in the *rpo*B core region was found resistant by both BACTEC and phage. All 18 isolates tested that were resistant to 2 μg/ml but susceptible to 4 μg/ml by phage displayed a wild type sequence for the *rpoB *core region. These 18 isolates were all susceptible by the BACTEC, suggesting their true susceptibility to rifampicin. The four isolates with discordant BACTEC and phage results at 4 or 10 μg/ml each harbored mutations predictive of resistance to rifampicin, two (both from the same patient) had Leu511Pro mutations and the other two had a Asp516Tyr and a Leu533Pro mutation.

**Table 2:** Mutations in the *rpoB *core region for 39 *M. tuberculosis *isolates found resistant to rifampicin by the phage assay.

Mutation	Number of isolates.
Ser531Leu	18
Asp516Val	9
Asp516Tyr	2
Leu511Pro	2
His526Tyr	2
Gln513Lys	1
Leu533Pro	1
Asp516Tyr	1
Ser531Leu & His526Tyr	1
Asp516Val & His526Tyr	1
No mutation	1

Total	39

### Minimal inhibitory concentration (MIC)

MIC's of the four isolates found resistant by phage at 4 or 10 μg/ml of drug but susceptible by BACTEC were determined by the colorimetric MTT method. The MIC of each isolate was found to be greater than 50 μg/ml compared to 2 μg/ml for the reference susceptible isolate (H37Rv) and all four isolates were judged to be truly resistant, in agreement with the phage and sequence data.

## Discussion

Several methods based on phage D29 replication have been reported for rapid drug resistance detection in *M. tuberculosis *isolates [4,6,13-17]. They differ with respect to the assay format, drug concentration and criteria for classifying isolates as resistant or susceptible. The reported studies indicate that phage replication technology offers the potential of a rapid, sensitive and specific test for detecting resistance to rifampicin. The methods reported initially required three to four days to complete and each isolate was tested in a single tube format, which is not convenient for screening large number of isolates. The phage method we have tested is a simple, low cost assay taking 48 hours to complete. For our study, the test was transferred to the Joint Clinical Research Centre (JCRC) in Kampala, Uganda, to assess its applicability and performance in a developing country setting.

When used with rifampicin concentrations at 4 or 10 μg/ml the phage assay was able to correctly detect all isolates classified as resistant to rifampicin by the BACTEC 460 (n = 35). Four additional isolates, found susceptible by Bactec 460, were identified as resistant by the phage assay. All 4 isolates were found to harbor mutations in the *rpo*B gene predictive of resistance, in addition they were found to have MIC's of over 50 μg/ml suggesting they were truly resistant. Thus the BACTEC 460 failed to detect 4/39 (10.2%) of rifampicin resistant isolates identified in the study. It could be postulated that the BACTEC culture was a mixed population, consisting of predominantly susceptible organisms but with a low level of (emerging) rifampicin resistant organisms. Retrospective analysis of BACTEC data showed that one isolate with a Leu533Pro mutation appeared to have a low level resistant population, yet the growth rate (change in growth indices) was not indicative of borderline resistance. Results of the other three isolates were clearly susceptible. It is of concern that the BACTEC was unable to satisfactorily detect four resistant isolates. These isolates were found resistant to other first line anti-tuberculosis drugs by BACTEC 460 and such patterns should prompt careful interpretation of the rifampicin result and/or additional testing.

Initial comparison between the phage assay and BACTEC methods suggested an estimated sensitivity and specificity for the phage assay of 100% and 96.5% respectively if the BACTEC was taken as the "gold standard". However, further analysis suggests that the BACTEC may record false negative results and might not, by itself, be an adequate method with which to evaluate new tests for drug resistance. In a previous study Albert *et al *reported a strain found negative by BACTEC 460 that was positive by a phage test that was subsequently found to harbor a mutation predictive of resistance [14]. BACTEC 460 has previously been considered as one of the gold standard tests for assessing susceptibility to anti-tuberculosis drugs. However, proficiency testing of traditional culture based methods undertaken by the WHO Supranational Laboratory Network indicates variable accuracy when testing susceptibility to rifampicin with an overall sensitivity and specificity of 97.2% and 96.8% respectively [1].

One isolate that was classified as resistant by both BACTEC 460 and the phage assay did not have a mutation in the *rpo*B core region. It has previously been reported that mutations in other regions of the gene may be responsible for resistance in a small proportion of *M. tuberculosis *strains [18]. Thus our results suggest the accuracy of the phage assay may be higher then that of molecular methods that are limited to screening the 81 bp genomic region of the rpoB gene.

When the phage assay was used with a drug concentration of 2 μg/ml 19 susceptible isolates were falsely identified as resistant. With this drug concentration the phage assay had a sensitivity of 100% but a specificity of 79.8%. When used at drug concentration of 4 or 10 μg/ml no false positives or false positive results were observed and the phage assay was more accurate than either the BACTEC 460 or the sequence analysis. Previous studies using mycobacteriophage D29 have applied a working concentration of 10 μg/ml [15,16] while a similar study in Spain applied a cut-off of 5 μg/ml [13]. The results presented here suggest a drug concentration of 4 μg/ml is adequate for this assay.

For implementation in resource poor settings such as sub-Saharan Africa new technology should preferably have low running costs and avoid need for major investment. The phage assay does not require sophisticated equipment other than that required for culture of tuberculosis. The estimated reagent and consumable costs, excluding labour and overheads, for testing a batch of 20 isolates for their susceptibility to rifampicin in a single 96 well plate format was 26 USD i.e. 1.3 USD per isolate.

## Conclusion

The phage assay can be used for screening of isolates for resistance to rifampicin, with high sensitivity and specificity when using a drug concentration of 4 μg/ml. The technique was easily transferable to a low-income country. The test is convenient, simple to perform and does not necessitate quantification of bacilli as required by other methods. We suggest the test may be useful in poorly resourced laboratories as a rapid screen to differentiate between rifampicin susceptible and potential MDR-TB cases.

## Competing interests

No author has competing financial or other interests. The London School of Hygiene & Tropical Medicine has an assignment & royalty agreement with a commercial company (Biotec Laboratories Ltd, UK) regarding IPR from previous studies on the use of phages for detection of bacteria.

## Authors' contributions

HT carried out MIC analysis, assisted with interpretation of phage and sequence data and contributed to writing the paper.

SO carried out the phage assay and BACTEC testing.

KM carried out sequence analysis.

MJ assisted with analysis and interpretation of BACTEC results.

FM carried out BACTEC testing.

KN carried out cost analysis.

SK carried out analysis of BACTEC results and assisted with cost analysis and drafting the article.

EJ participated in design and coordination of the study.

PS participated in design and coordination of the study and assisted in preparation of the manuscript.

JE participated in design and coordination of the study.

RM participated in design and coordination of the study.

KE assisted with interpretation of sequence and BACTEC data and contributed to writing the paper.

RMcN conceived the study, participated in its coordination, provided phage methodology/training, assisted with the cost analysis, assisted with interpretation of results, drafted the manuscript and is corresponding author.

## References

[B1] World Health Organisation (2004). Anti-tuberculosis drug resistance in the world: Report Number 3. The WHO/IUALTD global project on anti-tuberculosis drug resistance surveillance.. WHO/CDS/TB/2004343.

[B2] Morgan M, Kalantri S, Flores L, Pai M (2005). A commercial line probe assay for the rapid detection of rifampicin resistance in Mycobacterium tuberculosis: a systematic review and meta-analysis. BMC Infect Dis.

[B3] Traore H, Fissette K, Bastian I, Devleeschouwer M, Portaels F (2000). Detection of rifampicin resistance in Mycobacterium tuberculosis isolates from diverse countries by a commercial line probe assay as an initial indicator of multidrug resistance. Int J Tuberc Lung Dis.

[B4] Wilson SM, al-Suwaidi Z, McNerney R, Porter J, Drobniewski F (1997). Evaluation of a new rapid bacteriophage-based method for the drug susceptibility testing of Mycobacterium tuberculosis. Nat Med.

[B5] Jones WD, David HL (1971). Inhibition by rifampin of mycobacteriophage D29 replication in its drug-resistant host, Mycobacterium smegmatis ATCC 607.. Am Rev Respir Dis.

[B6] McNerney R, Kiepiela P, Bishop KS, Nye PM, Stoker NG (2000). Rapid screening of Mycobacterium tuberculosis for susceptibility to rifampicin and streptomycin. Int J Tuberc Lung Dis.

[B7] Telenti A, Imboden P, Marchesi F, Lowrie D, Cole S, Colston MJ, Matter L, Schopfer K, Bodmer T (1993). Detection of rifampicin-resistance mutations in Mycobacterium tuberculosis. Lancet.

[B8] McNerney R, Gillespie SH (2001). Micro-well phage replication assay for screening  mycobacteria for resistance to rifampicin and streptomycin. Antibiotic Resistance Methods and Protocols.

[B9] Siddiqi SH (1995). BACTEC 460 TB System. Product and Procedure Manual.

[B10] van Embden JD, Cave MD, Crawford JT, Dale JW, Eisenach KD, Gicquel B, Hermans P, Martin C, McAdam R, Shinnick TM (1993). Strain identification of Mycobacterium tuberculosis by DNA fingerprinting: recommendations for a standardized methodology. J Clin Microbiol.

[B11] Ohno H, Koga H, Kuroita T, Tomono K, Ogawa K, Yanagihara K, Yamamoto Y, Miyamoto J, Tashiro T, Kohno S (1997). Rapid prediction of rifampin susceptibility of Mycobacterium tuberculosis. Am J Respir Crit Care Med.

[B12] Abate G, Mshana RN, Miorner H (1998). Evaluation of a colorimetric assay based on 3-(4,5-dimethylthiazol-2-yl)-2,5-diphenyl tetrazolium bromide (MTT) for rapid detection of rifampicin resistance in Mycobacterium tuberculosis. Int J Tuberc Lung Dis.

[B13] Gali N, Dominguez J, Blanco S, Prat C, Quesada MD, Matas L, Ausina V (2003). Utility of an in-house mycobacteriophage-based assay for rapid detection of rifampin resistance in Mycobacterium tuberculosis clinical isolates. J Clin Microbiol.

[B14] Albert H, Trollip AP, Mole RJ, Hatch SJ, Blumberg L (2002). Rapid indication of multidrug-resistant tuberculosis from liquid cultures using FASTPlaqueTB-RIF, a manual phage-based test. Int J Tuberc Lung Dis.

[B15] Mani C, Selvakumar N, Kumar V, Narayanan S, Narayanan PR (2003). Comparison of DNA sequencing, PCR-SSCP and PhaB assays with indirect sensitivity testing for detection of rifampicin resistance in Mycobacterium tuberculosis. Int J Tuberc Lung Dis.

[B16] Simboli N, Takiff H, McNerney R, Lopez B, Martin A, Palomino JC, Barrera L, Ritacco V (2005). In-house phage amplification assay is a sound alternative for detecting rifampin-resistant Mycobacterium tuberculosis in low-resource settings. Antimicrob Agents Chemother.

[B17] Yzquierdo SL, Lemus D, Echemendia M, Montoro E, McNerney R, Martin A, Palomino JC (2006). Evaluation of phage assay for rapid phenotypic detection of rifampicin resistance in Mycobacterium tuberculosis. Ann Clin Microbiol Antimicrob.

[B18] Heep M, Brandstatter B, Rieger U, Lehn N, Richter E, Rusch-Gerdes S, Niemann S (2001). Frequency of rpoB mutations inside and outside the cluster I region in rifampin-resistant clinical Mycobacterium tuberculosis isolates. J Clin Microbiol.

